# Does Nocturnal Blood Pressure Matter in Retinal Small Vessels? A Systematic Review and Meta-Analysis of the Literature

**DOI:** 10.1007/s11906-025-01326-7

**Published:** 2025-01-18

**Authors:** Christina Antza, Smaro Palaska, Panagiota Anyfanti, Dimitris Triantis, Stavros Fyntrilakis, Yusuf ZiyaSener, Vasilios Kotsis

**Affiliations:** 1 Department of Internal Medicine, Aristotle University, Hypertension, Hypertension-24h ambulatory blood pressure monitoring center, Papageorgiou Hospital, Thessaloniki, Greece; 2 Thoraxcenter, Department of Cardiology, Erasmus MC University Medical Center, Rotterdam, The Netherlands

**Keywords:** Nocturnal hypertension, Dipping status, Non-dipping status, Nighttime hypertension, Retinal small vessels, Meta-analysis

## Abstract

**Purpose of the review:**

Τhe association between nocturnal blood pressure (BP) and alterations in the retinal microvasculature remains understudied, with few available studies to provide conflicting results. Therefore, we conducted a systematic review and meta-analysis to determine whether an association exists between retinal microvascular alterations and nocturnal BP patterns, determined by 24h ambulatory BP measurement.

**Recent findings:**

Our search concluded to 1002 patients (6 studies). A total of 3 studies (411 patients) were enrolled in the meta-analysis. Central retinal arteriolar equivalent found to be not different between patients with and without dipping status (mean differences [MD]: -0.01; 95% CI: -0.23 to 0.20; I²=0%; P < 0.610). Regarding central retinal venular equivalent, dippers showed significantly lower values compared to non-dippers (MD: -0.25; 95% CI: -0.47 to -0.03; I²=0%; P < 0.024). For the comparison between nighttime and daytime BP regarding the damage in small retinal vessels, we identified only 5 studies. Due to different evaluated outcomes as well as due to the heterogeneity of outcomes and different grouping of patients based on different BP cut-off values, these results couldn’t be analyzed quantitatively.

**Summary:**

In summary, this is the first effort to summarize evidence on the effects of day-to-night variation of BP on the retinal small vessels. According to the findings of the present systematic review and meta-analysis, non-dipping status may be associated with retinal venular dilatation, and elevated nighttime BP with retinal arteriolar narrowing. Further studies are warranted to elucidate the impact of nocturnal BP patterns in the retinal microvasculature.

**Supplementary Information:**

The online version contains supplementary material available at 10.1007/s11906-025-01326-7.

## Introduction

As a result of altered hemodynamics, hypertension induces structural and functional alterations in distant microvascular beds including the retinal microvasculature. Major technological advances have enabled the documentation of retinal microvascular alterations via digital retinal fundus photography, which is increasingly gaining place over classical, operator-dependent fundoscopic examination [1]. The traditional four-grade Keith-Wagener staging of hypertensive retinopathy remains the most widely cited classification system of retinal microvascular alterations that develop in response to increased blood pressure (BP) [2]. However, other vascular alterations not included in this grading system are more frequently encountered among hypertensive individuals, such as retinal vein occlusion (RVO) [3]. Noticeably, advanced stages of hypertensive retinopathy are rarely observed nowadays owing to relatively prompt diagnosis and effective hypertension management, and interest has gradually shifted over the clinical and prognostic significance of subtle retinal microvascular alterations. Changes in retinal vascular geometry, namely arteriolar narrowing and venular widening, as represented by central retinal arteriolar (CRAE) and venular equivalent (CRVE), as well as decreased arteriolar-to-venular ratio (AVR), have been associated with increased risk of cardiovascular disease (CVD) morbidity and mortality [4]. As consistently documented in several multitudinous studies, elevated BP is more closely and strongly linked to retinal arteriolar narrowing, which may even predict future onset of hypertension in initially normotensive individuals [5].

Ambulatory BP monitoring (ABPM) has incremental predictive value in terms of CVD risk, and offers a large amount of clinical information additional to conventional office BP measurements [6]. Importantly, 24-hour ambulatory BP monitoring remains at present the only widely available approach that enables assessment of nocturnal BP. Nocturnal BP phenotypes of particular clinical importance are elevated BP during nighttime, i.e., ≥ 120/70 mmHg, and abolishment of the normal BP drop during nighttime, i.e., a non-dipping BP pattern of less than 10% drop compared to daytime BP values. Nocturnal hypertension and/or non-dipping status are highly prevalent in conditions such as diabetes, obstructive sleep apnea, obesity, and chronic kidney disease, and their predictive value in terms of CVD risk is incremental not only in these high-risk patients but also further reflects in the general population [7]. More specifically, nighttime BP predicts CVD morbidity and mortality more effectively as compared to 24-hour, daytime and office BP levels, an effect probably attributed to the more standardized conditions under which BP is recorded during sleeptime [8, 9]. Furthermore, it has been overtly demonstrated in different clinical settings that adverse nocturnal BP phenotypes correlate with micro- and macrovascular injury, including arterial stiffness, left ventricular hypertrophy, carotid atherosclerosis, and albuminuria [10–12].

By contrast, the association between nocturnal BP and alterations in the retinal microvasculature remains understudied, with only few available studies that have provided divergent or even conflicting results. Therefore, we conducted a systematic review and meta-analysis to determine whether an association exists between retinal microvascular alterations and nocturnal BP patterns, determined by 24 h ABPM.

## Methods

This systematic review and meta-analysis is performed according to the Preferred Reporting Items for Systematic Reviews and Meta-Analyses (PRISMA) statement [13]. All research was conducted according to a protocol registered in the OSF database (available in https://osf.io/vsn9f/).

### Literature Search Strategy

A systematic search using Medline and Cochrane Library from inception to 30/09/2024 was performed to identify studies evaluating the impact of nighttime vs daytime BP as well as dipping vs non-dipping status on retinal small vessels. We used search terms that had been identified from initial scoping searches and target references. Search terms included, but not limited to, nocturnal hypertension, reverse dipping hypertension, dipping hypertension and retinal small vessels, central retinal artery equivalent, central retinal venous equivalent, arteriole-to-venule ratio, retinal vein occlusion. References of relevant studies and systematic reviews were perused and experts were contacted in order to identify any possible available study.

### Eligibility and Exclusion Criteria

We included original, cross-sectional or case-control or cohort studies examining the impact of nocturnal hypertension on retinal small vessels. The out of office BP measurements should have been defined by ABPM. Finally, full articles published in peer-reviewed journals were only included. The exclusion criteria were: (a) articles with full text in a language other than English, (b) studies that concern animals, (c) case reports, (d) systematic reviews, reviews and meta-analyses, (e) studies not including the effect on retinal small vessels.

### Data Extraction and Quality Assessment in Individual Studies

The searches’ results were imported in a reference management software (EndNote X7 for Windows, Thomson Reuters, Philadelphia, Pennsylvania). After removing the duplicate records, two reviewers (SP and JZS) screened for titles and abstracts independently and full texts were investigated for eligible studies. Differences between the two reviewers regarding study eligibility were resolved by a third reviewer (CA).

Study and population characteristics were extracted from each included study. Regarding study characteristics, the name of the first author, the year of publication, the country, were extracted. Also, the number of patients, the population, the primary outcome and details for the population were recorded.

### Quality Assessment

A total of 3 studies were eligible for the meta-analysis of the effect of dipping status on CRAE and CRVE. One of the studies was a randomized controlled trial, one was a cohort study and the other was a cross-sectional study. The quality of the studies and the risk of bias were assessed based on the type of study design. The QUADAS-2 tool was used to assess the risk of bias in the randomized controlled trial, while the Newcastle-Ottawa scale was used to assess the quality of the cohort and cross-sectional observational studies [14, 15].

### Data Synthesis – Statistical Analysis

Parametric variables were reported as mean ± standard deviation and parametric variables were presented as median (IQR). Categorical variables were presented as number (n) and percentage (%). Standard mean difference (SMD) with a 95% confidence interval (CI) was used to estimate the effect sizes of CRAE and CRVE. The statistical heterogeneity was evaluated using Q test (*p*-value < 0.1 indicating heterogeneity) and *I*^*2*^ statistic test (*I*^*2*^ > 30% or *p*-value < 0.1 indicating inconsistency). Fixed effects model was used to analysis due to the lower heterogeneity of the studies. Statistical analysis was performed using SPSS (Statistical Package for the Social Sciences) for Windows version 28.0 (SPSS Inc., Chicago, IL, USA).

## Results

### Search Results and Characteristics of Included Studies

**Fig. 1:**
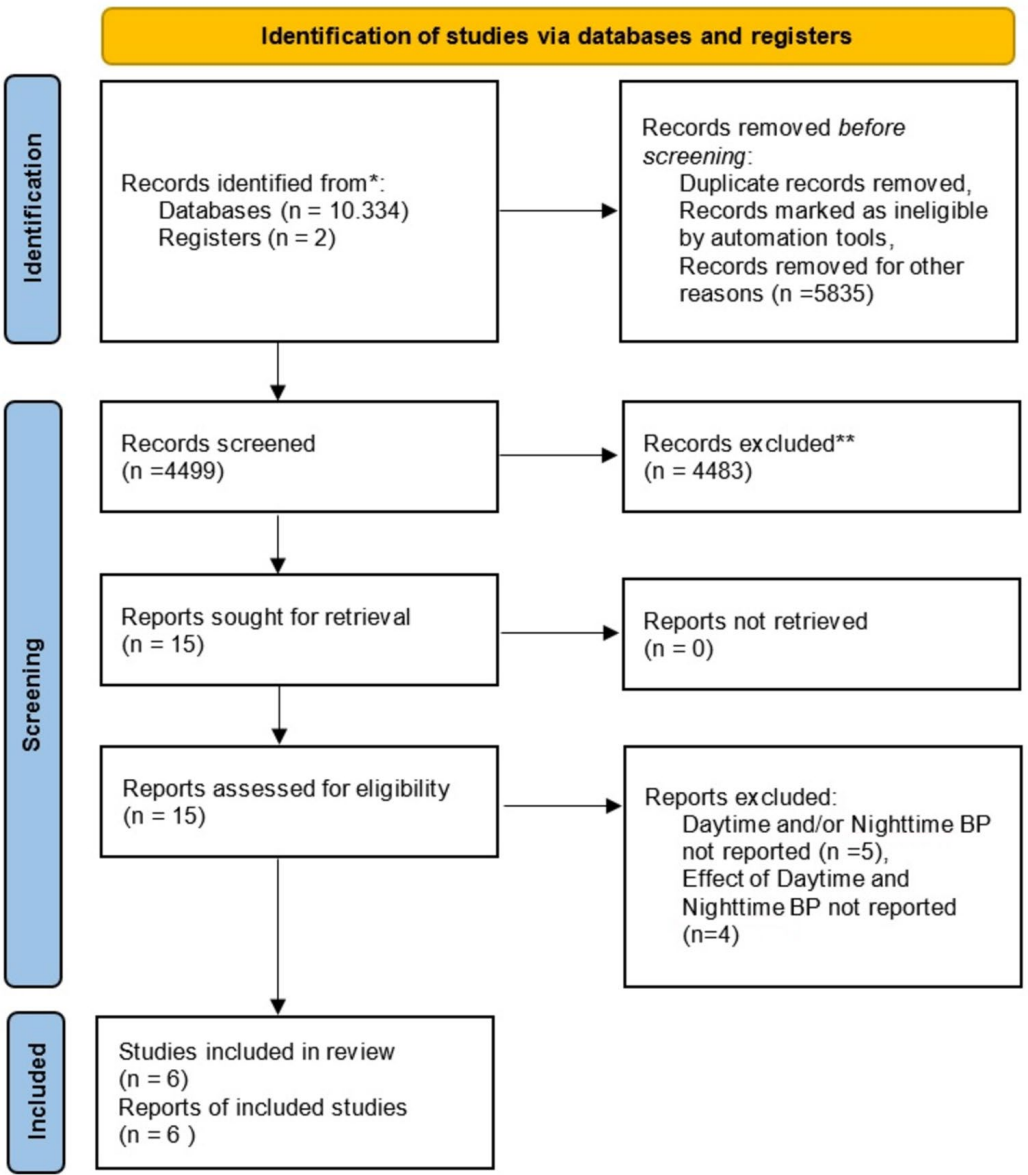
Summary of evidence search and selection

Our search concluded to 1002 patients. Only 6 studies met the inclusion criteria and finally were included to our systematic review [16–21]. Of them, only 3 (428 patients) fulfil the criteria to be included in the meta-analysis (Fig. 1) [17, 18, 21].

The characteristics of the 6 studies, included in our systematic review and meta-analysis, are shown in Table 1. Most of the studies were conducted in America, while the other studies were conducted in Australia, South Africa and East Asia, being published between 2010 and 2021. The design of the studies was mostly cross-sectional, and all studies include a relatively low number of patients (n < 150), except the one conducted by Fuchs et all (n = 448) [16]. As presented in Supplementary Table 1, the three studies included in the meta-analysis, were assessed either as low risk of bias [18] or as good [21] or very good [17] quality, based on QUADAS 2 or Newcastle Ottawa scale accordingly.

**Table 1:** Characteristics of the included studies

Author, Year of Publication	Region	Total population, Characteristics (age, sex)	Type of Study	Outcome	Comments
**Ali et al, 2019**	Australia (Melbourne)	N: 131 Age: 61.7 (14.5) 45% men (59)	Cross-sectional observational	Central Retinal Arteriolar Equivalent, Central Retinal Venular Equivalent	Caucasian population. Patients were recruited regardless of whether their hypertension was newly diagnosed or treated, and its duration was not assessed. Randomisation was not performed. Extra analysis for left ventricular hypertrophy.
**Fuchs et al, 2015**	South America (Brazil)	N: 448 subjects Age: 57.9 (12) 33% men (149)	Cross-sectional observational	Arteriolar, Venular, Arterio Venous Ratio, of inner and outer zone	Population from MONITOR study. Medical diagnosis of hypertension. Free from severe hypertension (office BP > 180/110mmHg), secondary hypertension, disabling chronic disease (cancer, liver cirrhosis, heart failure, unstable angina pectoris, mental disorders), and myocardial infarction or stroke within six months of enrollment. Patients were using on average of 1.4 ± 1.2 anti-hypertensive drugs, mostly ACE inhibitors (47%), diuretics (34%), β-blockers (34%), and amlodipine (14%).
**Smith et al, 2016**	South Africa (North-West)	N: 150 Age: Black 45.4 ± 6.9 White 49.3 ± 9.9 100% men	Cohort	Central Retinal Arteriolar Equivalent, Central Retinal Venular Equivalent	Population from SABPA study. Patients urban-dwelling black and white South African teachers between the ages of 20 and 65 years.
**Noh et al, 2021**	East Asia (South Korea)	N: 86 Age: Dipper 59.2 ± 1.7 Non dipper 61.2 ± 1.6 54,65% men	Retrospective	Branch retinal vein occlusion	Free from diagnosis of glaucoma, vitreous hemorrhage, retinal arterial occlusion, or diabetic retinopathy and prior history of focal/grid or panretinal photocoagulation, macular disease, or intraocular surgery, systemic medications for hypertension.
**Rao et al, 2016**	North America (North Carolina)	N: 40 Age: RVO 68,9 (8,4) NON–RVO 67,7 (7,6) 25% men	Cross-sectional observational	Retinal Vein Occlusion	Patients with a diagnosis of RVO. Patients with age > 45 years at the time of RVO. Patients free of known or suspected hypercoagulable states. The controls were matched by age and sex, masked to their BP level and dipping status
**Klein et al, 2010**	North America (USA, Canada)	N: 147 Age: 31.3 (9.3) 45,8% men (67)	Randomized controlled clinical trial, (cross sectional associations)	Central retinal arteriolar equivalent, Central retinal venular equivalent	Patients were ≥ 15 years old with 2–20 years of T1DM and onset before their 45th birthday. All were normotensive, albumin excretion rate < 20 µg/min on at least 2 of 3 timed overnight urine collections and had a normal or increased glomerular filtration rate ≥ 90 mL/min/1.73 m2. 285 subjects were randomized into one of the three treatment groups: losartan (an ARB), enalapril, or placebo.

In Table 2, the recorded means of systolic and diastolic BP during both daytime and nighttime are presented. Regarding systolic BP, all studies reported uncontrolled daytime and nighttime BP values, except the study of Klein et al [18]. This study provided the correlations in a normotensive population, which is clear from the mean BP values during ABPM.

**Table 2:** Recorded daytime and nighttime SBP and DBP (mmHg)

Author/ year	Daytime SBP (mean ± SD)	Daytime DBP (mean ± SD)	Nighttime SBP (mean ± SD)	Nighttime DBP (mean ± SD)
Ali et al/2019	N/A	N/A	N/A	N/A
Smith et al/2016	Black 141 ± 15 White 133 ± 10	Black 92 ± 10 White 85 ± 7	Black 127 ± 17 White 117 ± 13	Black 77 ± 12 White 70 ± 8
Fuchs et al/2015	135.5 ± 16.7	81.4 ± 12.3	125.2 ± 18.5	70.9 ± 12.5
Noh et al/2021	Dipper 149.3 ± 2.9 Non dipper 138.8 ± 2.3	Dipper 93.2 ± 2.0 Non dipper 86.4 ± 1.5	Dipper 127.3 ± 3.0 Non dipper 134.4 ± 2.1	Dipper 80.0 ± 2.3 Non dipper 83.2 ± 1.4
Rao et al/2016	RVO 146,2 ± 16,1 NON- RVO 139,6 ± 11,3	RVO 81 ± 7,9 NON- RVO 79,6 ± 9,8	RVO 138,4 ± 19,8 NON- RVO 123,1 ± 15,2	RVO 74,4 ± 11,9 NON- RVO 67,8 ± 8,6
Klein et al/2010	121.95 ± 9.66	75.44 ± 6.16	111.17 ± 9.66	65.34 ± 6.94

### Dipping vs Non-Dipping Blood Pressure Pattern

**Fig. 2:**
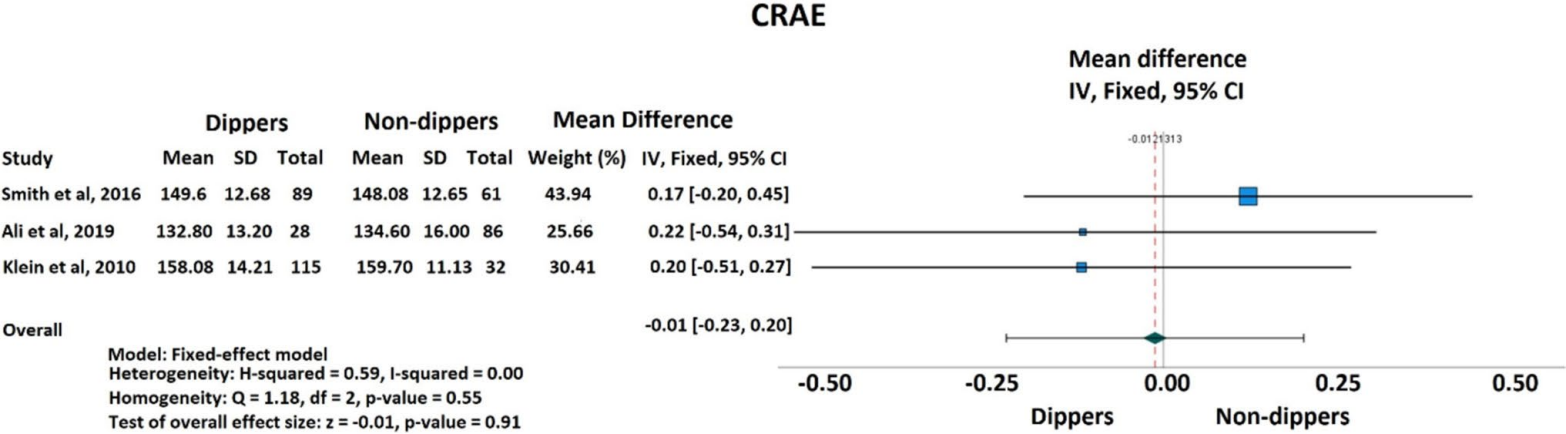
Forest plot of the effect of dipping versus non-dipping status on central retinal arteriolar equivalent

**Fig. 3:**
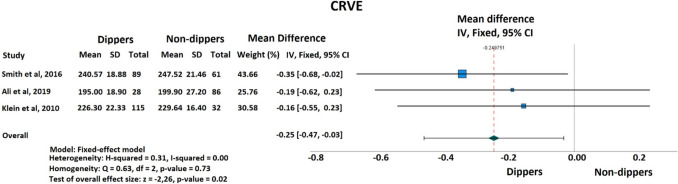
Forest Plot of the effect of dipping versus non-dipping status on central retinal venular equivalent

A total of 3 studies including 411 patients were enrolled in the meta-analysis. CRAE was established to be not different between patients with and without dipping status (mean differences [MD]: −0.01; 95% confidence intervals [11]: −0.23 to 0.20; *I²* = 0%; *P* < 0.610) (Fig. 2). Regarding CRVE, dippers showed significantly lower values compared to non-dippers (mean differences [MD]: −0.25; 95% confidence intervals [11]: −0.47 to −0.03; I² = 0%; *P* < 0.024) (Fig. 3).

### Nighttime vs Daytime Blood Pressure

For the comparison between nighttime and daytime BP regarding the damage in small retinal vessels, we identified only 5 studies [16–20]. The results of these studies are presented in Table 3. Due to different evaluated outcomes as well as due to the heterogeneity of outcomes and different grouping of patients based on different BP cut-off values, it was not possible to analyse these results quantitatively.

**Table 3:** The effect of daytime vs nighttime blood pressure on retinal small vessels

Author/ year	Outcome of Daytime SBP	Outcome of Daytime DBP	Outcome of Nighttime SBP	Outcome of Nighttime DBP
**Ali et al, 2019**	CRAE (mean ± SD) < 135mmHg 139.7 ± 12.7* ≥ 135mmHg 131.3 ± 16.0 CRVE(mean ± SD) < 135mmHg 200.0 ± 23.0 ≥ 135mmHg 198.4 ± 26.7	N/A	CRAE(mean ± SD) < 120mmHg 136.0 ± 11.8 ≥ 120mmHg 133.7 ± 16.3 CRVE(mean ± SD) < 120mmHg 193.1 ± 22.9 ≥ 120mmHg 200.7 ± 25.9	N/A
**Fuchs et al, 2015**	Per increase of 10mmHg Inner zone (Dif, 95CI)** Arteriolar −0.6 (−1.2 to 0.07) Venular 0.6 (−0.3 to 1.4) AVR −0.01 (−0.02 to −0.001)* Outer zone (Dif, 95CI)** Arteriolar −0.6 (−1.1 to −0.01)* Venular 0.2 (−0.7 to 1.0) AVR −0.003 (−0.01 to 0.004)	Per increase of 10mmHg Inner zone (Dif, 95CI)** Arteriolar −0.9 (−1.8 to 0.04) Venular 1.1 (−0.08 to 2.3) AVR −0.01 (−0.02 to 0.004)* Outer zone (Dif, 95CI)** Arteriolar −1.1 (−1.9 to −0.3)* Venular 0.8 (−0.3 to 2.0) AVR −0.01 (−0.02 to −0.001)*	Per increase of 10mmHg Inner zone (Dif, 95CI)** Arteriolar −0.7 (−1.3 to −0.08)* Venular 0.5 (−0.2 to 1.3) AVR −0.01 (−0.01 to 0.001) Outer zone (Dif, 95CI)** Arteriolar −0.5 (−1.0 to −0.03)* Venular −0.03 (−0.8 to 0.7) AVR −0.002 (−0.01 to 0.004)	Per increase of 10mmHg Inner zone (Dif, 95CI)** Arteriolar −1.0 (−1.9 to −0.1)* Venular 0.9 (−0.2 to 2.1) AVR −0.01 (−0.02 to −0.001)* Outer zone (Dif, 95CI)** Arteriolar −1.0 (−1.7 to −0.2)* Venular 0.2 (−1.0 to 1.3) AVR −0.01 (−0.02 to 0.004)
**Noh et al, 2021**	Macular Ischemia (β)*** − 0.157	Macular Ischemia (β)*** − 0.165	Macular Ischemia (β)*** 0.049	Macular Ischemia (β)*** 0.101
**Rao et al, 2016**	RVO (Dif,95%CI) 6.65 (− 2.3, 15.6)	RVO (Dif,95%CI) 1.4 (− 4.3, 7.1)	RVO (Dif,95%CI) 15.3 (3.9, 26.6)*	RVO (Dif,95%CI) 6.7 (0.1, 13.3)*
**Klein et al, 2010**	Per increase of 1mmHg CRAE (Dif ± SE)**** −0.29 ± 0.13* CRVE (Dif ± SE)**** 0.17 ± 0.20	Per increase of 1mmHg CRAE (Dif ± SE)**** −0.44 ± 0.21* CRVE (Dif ± SE)**** 0.03 ± 0.32	Per increase of 1mmHg CRAE (Dif ± SE)**** −0.27 ± 0.13* CRVE (Dif ± SE)**** 0.16 ± 0.19	Per increase of 1mmHg CRAE (Dif ± SE)**** −0.23 ± 0.19 CRVE (Dif ± SE)**** 0.23 ± 0.28

*CRAE* Central Retinal Arteriolar Equivalent, *CRVE* Central Retinal Venular Equivalent, *AVR* Arterio Venous Ratio, *RVO* Retinal Vein Occlusion, *p < 0.05, **adjusted for age, fellow vessel, gender, and duration of hypertension, ***Adjusted for age, sex, body mass index, current smoking status, and alcohol drinking status, ****Adjusting for site, baseline age, glycosylated hemoglobin level, and ambulatory pulse rate

Ali et al. [17], evaluated 131 patients, most of them presenting at least mild microvascular retinopathy. The results of this study showed that only subjects with a mean 24hour daytime systolic BP higher than 135 mmHg had a statistically significant reduced CRAE, but not CRVE. Nighttime BP or abnormal dipping status revealed no correlation either with CRAE or CRVE (p > 0.05). However, the results from Rao et al. [20] found no correlation with daytime BP and RVO, but a statistically significant difference for both systolic and diastolic nighttime BP [difference (95%CI): 15.3 (3.9, 26.6), 6.7 (0.1, 13.3) respectively, p < 0.05 for both]. In this study, the number of participants were extremely low; from 76 patients identified with RVO, finally only 22 elected to participate and underwent ABPM. Only one study evaluated the development of macular ischemia in patients with already established branch RVO [19]. In the univariate analysis, none of the daytime mean systolic and diastolic BP or nighttime mean systolic and diastolic BP showed association, with an exception for night-to-day systolic BP ratio(β = − 0.320, *P* = 0.003).

Regarding the effect of daytime and nighttime BP (per mmHg increase) on retinal small vessels, the systematic search concluded only to 2 studies [16, 18]. The first one, concluded that there is a statistically significant reduction in CRAE for every 1mmHg increase of the systolic and diastolic daytime BP, as well as in the systolic nighttime BP but not in the diastolic nighttime BP, after adjustment for important confounders such as site, age, glycosylated hemoglobin level, and ambulatory pulse rate. The highest impact on CRAE (difference ± SE: −0.44 ± 0.21) was observed for the1mmHg increase of the diastolic daytime BP. However, this study was conducted in a normotensive population, with a diagnosis of type 1 diabetes mellitus [18]. The second study, measured arteriolar and venular calibers by the microdensitometric method in 448 patients with hypertension. Per 10mmHg increase in nighttime systolic and diastolic BP we had a statistically significant decrease in inner and outer arteriolar caliber, adjusted for age, gender, fellow vessel, and duration of hypertension. Regarding the outer arteriolar caliber, also the increase of daytime systolic and diastolic BP was correlated with reduction. Inner AVR was also inversely associated to daytime and nighttime diastolic BP, while for the outer zone, the association of AVR was significant for daytime diastolic BP. Venular caliber was not associated with any of the BP measurements [16].

## Discussion

This systematic review and meta-analysis shows that nocturnal BP has an impact on retinal small vessels, although results may vary significantly according to the parameters under evaluation, either retinal (i.e., retinal arteriolar and venular widths, their ratio, or RVO) or ABPM-derived (dipping status, the degree of BP dipping, or absolute levels of 24-hour, daytime, or nighttime BP).More specifically, we conducted a meta-analysis demonstrating that categorization according to dipping status (i.e., dippers vs. non-dippers) is associated with altered retinal venular, yet not arteriolar, diameters. Furthermore, this systematic review provides some evidence that increased nighttime BP is associated with decreased retinal arteriolar width [16, 18], although these results were not unanimously reported [17]. However, due to divergent evaluated outcomes and different grouping of patients, we were not able to meta-analyze these data in order to quantitatively assess the impact of absolute levels of nighttime BP on retinal microvascular alterations. Finally, there is limited evidence that increased nighttime BP correlates with RVO [20], although it does not seem to affect the development of macular ischemia in patients with already established branch RVO [19].

Our finding that non-dippers exhibit increased values of CRVE is in line with a large body of evidence supporting that larger CVRE is associated with increased CVD risk and adverse CVD outcomes. In the Atherosclerosis Risk in Communities (ARIC) Study, wider retinal venules as well as narrower retinal arterioles conferred long-term risk of mortality and ischemic stroke in both sexes, and increased risk of coronary heart disease in the female population [22]. More recent analysis from the ARIC Study showed that CRVE widening was further associated with larger left ventricular size, higher prevalence of left ventricular hypertrophy, and worse measures of diastolic and systolic function over the mean 16-year follow-up period, and the same was observed for CRAE narrowing [23]. However, the most concrete evidence regarding the clinical significance of retinal venular dilatation comes from several population-based studies that show an association with the progression of cerebral small vessel disease, increased risk of lacunar stroke, ischemic and hemorrhagic stroke [22–25]. These data reinforce the hypothesis that the retinal microvasculature provides a direct “window” to the brain, further suggesting that increased CRVE may serve as an early marker of cerebral vascular disease.

Whereas venular width was associated with dipping status, the present meta-analysis does not support such an association with CRAE. Oppositely, our systematic review points towards an association of CRAE, yet not with CRVE, with absolute nighttime BP levels. These divergent findings should be considered as complementary, rather than contradictory, and imply a different prognostic role for each retinal microvascular diameter (arteriolar or venular). Epidemiological data indicate that non-dipping pattern confers a higher risk of future stroke [26, 27]. As mentioned above, retinal venular widening may be used as an early indicator of future risk of stroke. Taken together, it could be hypothesized based on our results that the non-dipping pattern provokes cerebral vascular injury, that can be non-invasively assessed through the study of retinal venular width at pre-symptomatic stages.

On the other hand, there is an inherent relationship between absolute levels of BP and retinal arteriolar diameters. Elevated levels of both conventional and 24h ambulatory BP primarily affect the retinal arterioles, especially during early stages of hypertension, and have been associated with decreased retinal arteriolar width [28]; our study further reinforces this evidence by suggesting an inverse association between CRAE and nighttime BP levels as well [16, 18]. This association was not reproduced in a study that dichotomized nighttime systolic BP according to the conventional cut-off threshold of 120 mmHg [17]; therefore, we were not able to meta-analyze these studies. Remarkably, the association between retinal arteriolar width and BP is bi-directional. The strong impact of elevated BP on the retinal arterioles has long been known and characterizes the first stage of hypertensive retinopathy according to the Keith-Wagener classification. [2] Vice versa, retinal arteriolar narrowing may predict future onset of hypertension to a larger extent than retinal venular width, and supports the concept that generalized microvascular remodeling, as observed in the retinal microvasculature, precedes the onset and progression of hypertension [29].

Regardless of the causality of the observed associations, our study provides some important clinical implications. 24h ABPM is being increasingly recognized as the method of choice, whenever possible, for establishing the diagnosis of hypertension and for subsequent BP monitoring [30]. BP recording throughout the whole 24h period offers the unique advantage of diagnosing nocturnal hypertension and identification of dipping status. Based on the results of the present study, identification of elevated nighttime BP levels as well as non-dipping profile warrants a thorough screening for pre-symptomatic injury in divergent vascular beds, including the retinal microvasculature. Vice versa, phycisians should be alert upon identification of subtle microvascular alterations in the retinal microvasculature that may imply altered day-to-night BP variation, and consider implementation of 24h ABPM. Finally, the results of our study generate further hypotheses regarding the potential effects of chronotherapy, i.e., treatment aiming at restoring the nocturnalcircadian BP rhythm, on the reversal of microvascular injury related to elevated nighttime BP.

Limitations of the present study that downgrade the certainty of the provided evidence are inherent to the limitations of the included studies, which were in their majority observational, non-randomized and applied different entry criteria and outcome measures. Further limitation of the study is that the included studied use different methodology to assess retinal vasculature. Hence, owing to the heterogeneity of outcomes and the different synthesis of study populations, it was not feasible to meta-analyze data regarding the association between CRAE and nighttime BP. We were able to perform a meta-analysis of three available studies regarding the association between dipping status and retinal microvascular diameters; however, one of these studies was conducted in a distinct population of normotensive patients with type 1 diabetes mellitus [18]. Although this does not negate the impact of dipping status on the retinal microvasculature, it further highlights the need for appropriately designed studies to evaluate the association between nocturnal BP and retinal microvascular alterations.

In summary, this is the first effort to summarize evidence on the effects of day-to-night variation of BP on the retinal small vessels. According to the findings of the present systematic review and meta-analysis, non-dipping status may be associated with retinal venular dilatation, and elevated nighttime BP with retinal arteriolar narrowing. Further studies are warranted to elucidate the impact of nocturnal BP patterns in the retinal microvasculature. However, these results underscore the clinical significance of nocturnal BP as a mediator of generalized vascular impairment, and call for a more careful evaluation of patients who present with abnormal nighttime BP in the context of subclinical vascular injury.

## Key References

- Iorga RE, Costin D, Munteanu-Dănulescu RS, Rezuș E, Moraru AD. Non-Invasive Retinal Vessel Analysis as a Predictor for Cardiovascular Disease. J Pers Med. 2024;14(5).
  - ○ Vessel Analysis as a Predictor for Cardiovascular Disease. J Pers Med. 2024;14(5). This study states the importance of retinal vessel analysis in cardiovascular disease risk.
- Panagiotakos D, Antza C, Kotsis V. Ambulatory and home blood pressure monitoring for cardiovascular disease risk evaluation: a systematic review and meta-analysis of prospective cohort studies. J Hypertens. 2024;42(1):1–9.
  - ○ This study suggests that ambulatory blood pressure monitoring offers significant advantages in blood pressure measurement over home and office blood pressure measurements.
- Anyfanti P, Malliora A, Chionidou A, Mastrogiannis K, Lazaridis A, Gkaliagkousi E. Clinical Significance of Nocturnal Hypertension and Nighttime Blood Pressure Dipping in Hypertension. Curr Hypertens Rep. 2024;26(2):69–80.
  - ○ This study is of great importance, because it demonstrates that nocturnal hypertension and non-dipping pattern are predictive factors for cardiovascular problems. It also shows that the diagnosis of abnormal nocturnal blood pressure patterns is based on ambulatory blood pressure monitoring.
- Staplin N, de la Sierra A, Ruilope LM, Emberson JR, Vinyoles E, Gorostidi M, et al. Relationship between clinic and ambulatory blood pressure and mortality: an observational cohort study in 59 124 patients. Lancet. 2023;401(10393):2041-50.
  - ○ This study shows that nocturnal blood pressure is superior as a predictive tool for cardiovascular death.

## Supplementary Information

Below is the link to the electronic supplementary material.

Supplementary Material 1 (DOCX 13.6 KB)

## Data Availability

No datasets were generated or analysed during the current study.

## Abbreviations

BP: Blood Pressure
RVO: Retinal Vein Occlusion
CRAE: Central Retinal Arteriolar Equivalent
CRVE: Central Retinal Venular Equivalent
AVR: Arteriolar-to-Venular Ratio
CVD: Cardiovascular Disease
ABPM: Ambulatory BP Monitoring
SMD: Standard Mean Difference
CI: Confidence Interval
MD: Mean Differences
