# Supplementary material for: Does Nocturnal Blood Pressure Matter in Retinal Small Vessels? A Systematic Review and Meta-Analysis of the Literature
Source: Curr Hypertens Rep. 2025 Jan 18;27(1):9. doi: 10.1007/s11906-025-01326-7 (PMC11742766; doi:10.1007/s11906-025-01326-7)
Supplement: Supplementary file 1 — Supplementary Material 1 (DOCX 13.6 KB) [file 11906_2025_1326_MOESM1_ESM.docx]

| **Table- S1. Assessment of the quality of enrolled studies** | | | | | |
| --- | --- | --- | --- | --- | --- |
| **QUADAS-2** | **Klein et al.** | **Newcastle-Ottawa Scale (Cohort study)** | **Smith et al.** | **Newcastle-Ottawa Scale (Cross-sectional study)** | **Ali et al.** |
| **Risk of Bias** |  | **Selection (Max 4 stars)** |  | **Selection (Max 5 stars)** |  |
| Patient selection | **☺** | Representativeness of the exposed cohort | * | Representativeness of the cases | * |
| Index test | **☺** | Selection of the non-exposed cohort | * | Sample size | - |
| Reference standard | **☺** | Ascertainment of exposure | * | Non-response rate | * |
| Flow and timing | **☺** | Demonstration that outcome of interest was not present at start of study | * | Ascertainment of the screening/surveillance tool | * |
| **Applicability Concerns** |  | **Comperability**  **(Max 2 stars)** |  | **Comperability**  **(Max 2 stars)** |  |
| Patient Selection | **☺** | Comperability of cohorts on the basis of the design or analysis | ** | The potential confounders were investigated by subgroup analysis or multivariable analysis | ** |
| Index test | **☺** | **Outcome (Max 3 stars)** |  | **Outcome (Max 3 stars)** |  |
| Reference standard | **☺** | Assessment of outcome | * | Assessment of outcome | ** |
|  |  | Was follow-up long enough for outcomes to occur | * | Statistical outcome | * |
|  |  | Adequacy of follow up of cohorts | * |  |  |
| **Final Result** | **Low risk of bias** |  | **Good quality (9/9)** |  | **Very good (9/10)** |
